# Emerging methods in botanical DNA/RNA extraction

**DOI:** 10.1002/aps3.11530

**Published:** 2023-06-16

**Authors:** Nora Mitchell, Edward V. McAssey, Richard G. J. Hodel

**Affiliations:** ^1^ Department of Biology University of Wisconsin–Eau Claire Eau Claire Wisconsin USA; ^2^ Department of Ecology and Evolutionary Biology University of Connecticut Storrs Connecticut USA; ^3^ Department of Botany National Museum of Natural History Washington D.C. USA; ^4^ Data Science Lab, Office of the Chief Information Officer, Smithsonian Institution Washington D.C. USA

Analyses of nucleic acids (DNA and RNA) have become a staple tool for botanists to answer questions across a wide variety of disciplines, ranging from population genetics to biogeography, ecology, development, microbiology, physiology, and phylogenetics. The rise of “next‐generation” or “high‐throughput” sequencing in particular has resulted in reduced sequencing costs and an explosion in the number of botanical studies using DNA or RNA data (Egan et al., 2012). Yet, the crucial step of extracting these nucleic acids from plant tissues can be extremely difficult and is often overlooked or under‐emphasized. Although there are many options for nucleic acid kits and nearly countless papers (over 22,000 at the time of this special issue) referencing a “modified” version of the Doyle and Doyle (1987) cetyltrimethylammonium bromide (CTAB) extraction protocol, taxon‐specific difficulties render many of these methods ineffective. Troubleshooting the extraction step remains a major sink of researchers' time and energy, potentially acting as a barrier to downstream analyses and answering fundamental botanical questions.

Difficulties in nucleic acid extraction arise due to factors such as the diversity and volume of secondary metabolites expressed by plants (Varma et al., 2007), degradation during storage (Pyle and Adams, 1989), contamination from DNA of organisms in the plant microbiome (Trivedi et al., 2022), and the need for high‐molecular‐weight nucleic acids for downstream analyses (Pollard et al., 2018). Addressing these issues requires knowledge of both the underlying chemistry involved during each step of the extraction process and the requirements of the isolated product. The 12 papers in this special issue, “Emerging Methods in Botanical DNA/RNA Extraction,” highlight the current state of knowledge in nucleic acid extractions, including both the key challenges and creative innovations that have been developed to circumvent these difficulties to address a variety of exciting botanical questions.

## CTAB modification

The most notable DNA extraction protocol for plants is the CTAB‐based approach of Doyle and Doyle (1987). However, researchers commonly refer to a “modified CTAB” approach, where various modifications address difficulties in extraction that are often taxon‐specific and highly varied, but often without detailing what aspects of the protocol were adjusted. To better understand these alterations, the first paper in this issue (Schenk et al., 2023) reports the results of a literature survey and summary of these modified CTAB protocols. Schenk et al. (2023) report and provide recommendations for modifications to eight steps in the CTAB protocol: tissue preparation, suspension, lysis, isolation, cleaning, elution, secondary cleanup, and quantification, with explanations as to why each step may require modification. Additionally, they provide four supplementary protocols as appendices, which detail the alterations to the lysis and/or extraction steps. This review will allow researchers to troubleshoot their own DNA extractions while also promoting repeatability and transparency.

## Recalcitrant plants

Many modifications to nucleic acid extraction are designed for “recalcitrant” plants, or species whose chemical composition or anatomy make extraction much more difficult. Three articles in this special issue address specific ways to improve nucleic acid extraction from plants that prove recalcitrant for different reasons.

Recalcitrant plant species may contain high amounts of hydrolytic enzymes that exhibit nuclease activity, as do silica‐dried plant tissues. G. Johnson et al. (2023) develop protocols that demonstrate the utility of ethanol, rather than silica gel, as a desiccant prior to DNA extractions to increase DNA yield and quality. Ethanol may be advantageous as it can act to inhibit hydrolytic enzyme activity and allow for easier cell wall disruption. The authors compare the utility of ethanol methods using three examples: direct ethanol collection in Vitaceae, ethanol pretreatment of silica‐dried tissues in the recalcitrant mangrove species *Rhizophora mangle* (Rhizophoraceae), and ethanol pretreatment of herbarium specimens from 30 taxa representing nine plant families. G. Johnson et al. (2023) report that ethanol increases the quality and quantity of extracted DNA, especially with appropriate proteinase digestion and other treatments prior to lysis.

To address plants that are recalcitrant due to sclerophylly, Jones et al. (2023) investigate the effects of mechanical tissue disruption, storage time, and leaflet age on DNA extraction quality and concentration using the thick and rigid leaves of the cycad genus *Encephalartos*. In cycads, recalcitrant leaf tissue yielded greater concentrations of DNA when tissue was manually disrupted using a mortar and pestle vs. bead‐based disruption, although DNA purity was unaffected by the disruption method. Tissue stored in silica gel also yielded greater DNA concentrations, but with no impact on purity, when compared to freshly collected tissue. The time of storage in silica gel ranged from less than a year to a decade, with no significant effect on resulting DNA concentration. Finally, Jones et al. (2023) reveal that both senescing and young leaflets could adequately yield sufficient, pure DNA for downstream applications.

The extraction of RNA from plant tissues can also prove extremely challenging, especially given the presence of polyphenols and polysaccharides found in recalcitrant woody species. Hadi and Stacy (2023) test the effectiveness of four RNA isolation kits and three other protocols (along with modifications) on young leaf tissue from three *Metrosideros* species (Myrtaceae) that differ in anatomical and chemical makeup. They use absorbance ratios, RNA integrity number (RIN), and the success of downstream RNA‐Seq analyses to compare the effectiveness of nine protocols. Hadi and Stacy (2023) report that only one popular isolation kit was effective in these taxa, and they further optimize the protocol, resulting in high‐quality RNA suitable for downstream RNA‐Seq.

## Effect of different storage methods on DNA extraction

The collection and storage of plant tissue can also impact the optimization of nucleic acid extraction. As many botanists conduct research in field settings, the ability to rapidly extract DNA in the field can be a great advantage, especially for plant tissues that do not store well. In this special issue, Selz et al. (2023) develop a protocol to use polymeric microneedle patches to rapidly collect DNA directly from leaf tissue in the field. The microneedle patches, originally developed for drug delivery, can be produced in the lab and taken to the field. After a brief application to a leaf surface, the microneedle patches only require elution in ultrapure water and cleaning via spin column before the DNA is ready for downstream applications. The microneedle patch approach was tested using standard nuclear and chloroplast barcoding loci and produced similar results to commercial kits in its ability to barcode species represented in the National Center for Biotechnology Information's GenBank database. This approach offers researchers a flexible and practical option for extracting DNA in remote locations.

Although immediate nucleic acid extraction is useful for field collection, the use of specimens housed in long‐term storage provides exciting opportunities to investigate historical and contemporary questions using existing specimens. Herbarium specimens represent a rich resource to explore questions related to climate change and evolution, as well as providing access to potentially rare or even extinct taxa. DNA extraction from herbarium specimens is feasible, but needs to be optimized to account for chemical changes in storage as well as to maximize yield from small amounts of tissue so that specimens may be used for additional purposes in the future (Besnard et al., 2018; Funk, 2018). In this issue, Gouker et al. (2023) optimize a previously published protocol to study the effects of DNA extraction procedure, species, and specimen age on DNA yield. While a commercial kit outperformed other tested methods in terms of yield for specific taxa, all methods produced enough DNA for most subsequent experiments. Interestingly, across the entire data set, all tested methods (commercial kit, acetone, and CTAB) produced no trend in terms of the effect of age on DNA concentration within both old (before 1960) and new (after 1960) herbarium samples, although species‐specific differences between old vs. new specimens existed.

Different storage methods require unique modifications to extraction protocols and may also affect the quality of nucleic acids. Two articles in this special issue compare storage methods and optimize protocols for DNA extraction. To explicitly compare storage methods, Carey et al. (2023) experimentally test how various additives and incubation considerations during the CTAB lysis step affect extraction from both silica‐dried and herbarium leaf tissues using four genera. Based on their results, they recommend using shorter and cooler incubation periods during the lysis step and using fresh silica‐dried leaf tissue (over herbarium tissue) when possible.

Recently, researchers have moved toward storing frozen DNA samples along with typical herbarium samples. McAssey et al. (2023) creatively use a data set—the Hawaiian Plant DNA Library, collected from 1994–2019—with both frozen DNA accessions and herbarium sheets to compare each storage method for use in downstream sequencing applications. A comparison of paired samples revealed that DNA obtained from herbarium specimens was significantly more fragmented than DNA stored in a freezer, and consequently produced lower‐quality chloroplast assemblies. Nevertheless, the recovery of nuclear genes from short‐read high‐throughput sequencing was not affected by the storage method; instead, the age of the specimen had a more substantial impact on gene recovery. Plant tissues in herbarium collections will continue to be a great resource for DNA extraction, but adding frozen DNA storage as a standard practice in herbaria will improve our ability to use DNA evidence to study biodiversity in the future.

## Environmental DNA (eDNA)

Environmental DNA (eDNA) has been used to assess the taxonomic diversity present in various environmental sources, such as air samples, organismal products (like feces or honey), water, and soils (M. D. Johnson et al., 2023), as well as the diversity present in the plant microbiome, i.e., the biotic communities found in or on different plant tissues (Trivedi et al., 2022). The combined use of high‐throughput sequencing with eDNA (eDNA metabarcoding, or metagenomics) is further expanding the potential to survey and identify entire communities (Deiner et al., 2017). In additional exciting developments, these technologies to describe biodiversity are being applied to diverse taxa and tissue types. For example, Guillen‐Otero et al. (2023) use ITS and 18S rRNA metabarcoding to characterize the fungal biodiversity that exists on ferns and lycophytes. Interestingly, this study was conducted on a relatively challenging tissue—root tissue—to describe the diversity of arbuscular mycorrhizal fungi. The communities measured in ferns and lycophytes provide important insights into the potential fungal composition found in the most recent common ancestor shared with angiosperms.

## Long‐read and high‐molecular‐weight sequencing

Long‐read platforms offer the capability of generating sequences of tens or even hundreds of thousands of base pairs. These platforms, developed by companies such as PacBio (Menlo Park, California, USA) and Oxford Nanopore Technologies (Oxford, United Kingdom), are sometimes referred to as “third‐generation” technologies. Long reads have helped to facilitate the transition from the assembly of short reads (50–200 bp in length) to more efficient means of whole genome sequencing, with nearly 800 plant genomes sequenced (Marks et al., 2021). Moreover, short‐read sequencing is challenging for taxa that are heavy in transposable elements (TEs), so long‐read technology can also expand the taxonomic breadth of genome sequencing (Shahid and Slotkin, 2020). These technologies require high‐molecular‐weight DNA (fragments of 50 kbp or longer), which is generally obtained via modification of protocols that are designed for obtaining shorter fragment lengths from DNA samples with little degradation.

To that end, De La Cerda et al. (2023) present a series of straightforward modifications (including mortar and pestle vs. beads and cut vs. intact pipette tips) to standard protocols to use nanopore sequencing to enable plant evolutionary studies in *Calochortus*, which has relatively large genomes. Furthermore, the authors modified a sodium dodecyl sulfate–based DNA extraction protocol to promote less‐contaminated DNA, which is critical for long‐read sequencing technologies. Any modifications that can improve DNA length and quality result in a more efficient sequencing run, which is of primary concern for researchers working in clades that are known for having taxa with large genomes.

In a complementary fashion, Kang et al. (2023) performed a series of DNA extraction modifications to optimize long‐read sequencing in a diverse set of taxa spanning 18 orders. Testing a protocol in diverse taxa is especially important as long‐read sequencing continues to be a method of choice for biodiversity studies. The authors developed a protocol that combines a nuclear extraction method (to reduce organellar DNA) with a CTAB method, including important modifications to reduce problems associated with secondary metabolites. They compared their protocol to a commercial kit and found that the novel protocol resulted in longer DNA fragments and less contamination. Importantly, Kang et al. (2023) report that while their protocol recovered a substantial fraction of long reads, there was significant variability among taxa, presumably due to varying levels of secondary compounds naturally found in leaves.

Many fern lineages have notoriously large genomes, which necessitates modifications to DNA extraction protocols to ensure sufficient quantities of high‐molecular‐weight DNA to complete long‐read sequencing. Xie et al. (2023) presented two modifications to standard CTAB DNA extraction protocols that increase DNA yield: a strategy to prevent DNA shearing and a nuclei isolation approach that results in substantially larger quantities of extracted DNA. Although it is established that preventing DNA shearing improves long‐read sequencing performance (Gong et al., 2019), Xie et al. (2023) introduce a nuclei isolation step that, while requiring more input tissue, generates an order of magnitude more DNA per extraction than standard approaches. Two genomes have been sequenced using the protocol modifications developed by Xie et al. (2023), and both have read length N50 scores >14 kbp (Wickell et al., 2021; Rahmatpour et al., 2023).

Overall, the articles in this special issue highlight the novel uses of nucleic acid data for answering botanical research questions, aspects of cutting‐edge technologies used to generate these data, and questions to consider in the all‐important step of nucleic acid extraction. The diversity of taxon‐specific concerns, storage methods, source materials, and sequencing technologies require optimization and creative solutions for obtaining both DNA and RNA molecules. We hope that this special issue will help readers to improve their own protocols, expand their toolkits, and consider additional research frontiers enabled by nucleic acid data.

## AUTHOR CONTRIBUTIONS

N.M. prepared the first draft of the manuscript. All authors provided select article summaries and reviewing and editing assistance and approved the final version of the manuscript.
